# Is the Water Footprint an Appropriate Tool for Forestry and Forest Products: The Fennoscandian Case

**DOI:** 10.1007/s13280-013-0380-z

**Published:** 2013-02-19

**Authors:** Samuli Launiainen, Martyn N. Futter, David Ellison, Nicholas Clarke, Leena Finér, Lars Högbom, Ari Laurén, Eva Ring

**Affiliations:** 1 Joensuu Research Unit, Finnish Forest Research Institute, P.O. Box 68, 80101 Joensuu, Finland; 2 Department of Aquatic Sciences and Assessment, Swedish University of Agricultural Sciences, P.O. Box 7050, Vallvägen 3, 750 07 Uppsala, Sweden; 3 Institute for World Economics, Research Centre for Economic and Regional Studies, Hungarian Academy of Sciences, Országház utca 30, Budapest, 1014 Hungary; 4 Norwegian Forest and Landscape Institute, P.O. Box 115, 1431 Ås, Norway; 5 Skogforsk, Uppsala Science Park, 751 83 Uppsala, Sweden

**Keywords:** Environmental communication, Forests, Hydrologic cycle, Sustainability, Water footprint, Water use efficiency

## Abstract

The water footprint by the Water Footprint Network (WF) is an ambitious tool for measuring human appropriation and promoting sustainable use of fresh water. Using recent case studies and examples from water-abundant Fennoscandia, we consider whether it is an appropriate tool for evaluating the water use of forestry and forest-based products. We show that aggregating catchment level water consumption over a product life cycle does not consider fresh water as a renewable resource and is inconsistent with the principles of the hydrologic cycle. Currently, the WF assumes that all evapotranspiration (ET) from forests is a human appropriation of water although ET from managed forests in Fennoscandia is indistinguishable from that of unmanaged forests. We suggest that ET should not be included in the water footprint of rain-fed forestry and forest-based products. Tools for sustainable water management should always contextualize water use and water impacts with local water availability and environmental sensitivity.

## Introduction

Water is a precious and unevenly distributed resource that must be used in a sustainable manner. Population growth and rapid economic development increase pressures on global fresh water resources through growing demand for agricultural production, industrial goods, and bioenergy. In many regions, freshwater availability and quality issues already impact human well-being, mediate economic growth, and contribute to loss of ecosystem functions and biodiversity (Vörösmarty et al. 2010). The contrast between water-abundant and water-scarce areas is likely to further increase due to anthropogenic climate change (Held and Soden 2006; Bengtsson 2010). Societal recognition of the importance of sustainable water use has led to the establishment of numerous methods and initiatives to understand and measure human appropriation of global freshwater resources. Sustainable water use and environmental responsibility are of particular importance to the forest sector, which is a large user of fresh water both in its direct operations such as fiber processing and indirectly in tree growth for wood production (NCASI 2009, 2010; StoraEnso 2011; UPM 2011; Eriksson et al. 2011; Wiegand et al. 2011). Sustainability of fresh water use can be understood in at least two ways. Sustainability can be defined in terms of relative fresh water availability (quantity), suggesting that sustainable water use should not exceed available, renewable supply. It can also be defined in terms of potential water quality degradation or negative impacts on ecosystem service delivery. In the forest sector, sustainable water use means applying water and energy efficient processes and technologies, efficient waste water purification methods and limiting consumption to levels supported by local water resources. In forestry, water sustainability means minimizing negative impacts due to changes in quantity and/or quality of surface and ground waters. So as to measure sustainability, comparative performance and to assess water-associated business risks and communicate with customers and other stakeholders, the forest sector has been actively involved in developing water use metrics (NCASI 2009, 2010; UPM 2011).

In a recent summary, 16 different “water footprinting” tools for corporations or organizations were identified (WBSCD 2010). Currently, the most recognized method, hereafter referred as the WF, is that developed by the Water Footprint Network (Hoekstra et al. 2011). The WF is based on virtual water content (Allan 1998) which aggregates fresh water consumption over a production chain, and purports to represent the total amount of water consumed to produce a product or a service. It provides a framework for measuring human appropriation of fresh water and addresses issues of water scarcity, water use efficiency, and water use sustainability. The WF can in principle be applied from catchment to global scales, and be used to identify business, process or product level water consumption (Hoekstra et al. 2011). The WF divides freshwater use into green, blue, and gray components (Hoekstra et al. 2011). Blue water includes surface and ground water while green water represents rain water and water in the root zone that eventually contributes to plant growth through evapotranspiration (ET) (Falkenmark and Rockström 2006). Consequently, the blue water footprint represents consumption of surface and groundwater resources and the green water footprint is typically assumed to be equal to water evaporated when producing a unit of product or service. The gray water footprint represents the volume of fresh water for which water quality is degraded during production of a unit of product or service (Hoekstra et al. 2011).

This article is motivated by several concerns. First, according to WF case studies, forest ET is by far the largest component of the water footprint of forest-based products (van Oel and Hoekstra 2010, 2012; StoraEnso 2011; UPM 2011), similarly to that of agricultural goods (Riddout et al. 2009; Riddout and Pfister 2010; SABMiller and WWF 2010; Mekonnen and Hoekstra 2011) and agricultural bioenergy (Gerbens-Leenes et al. 2009; Pfister and Hellweg 2009; Jeswani and Azapagic 2011; Gheewala et al. 2011). Therefore, it is necessary to explore whether ET is correctly included in the WF taking into account its natural role in the hydrologic cycle. If this is not the case, it is possible that conclusions about water use of forestry and forest-based products are incorrect. We are not aware of any studies that adequately address these issues for forest-based products, although they have been touched upon in some studies of agricultural bioenergy (Gheewala et al. 2011). Second, no studies exist that have critically and objectively considered the applicability of the WF for forestry.

In this article, we critically evaluate whether the WF is a suitable tool for water footprinting of forestry and forest-based products using forestry in the Fennoscandia as a case example. First, we briefly describe the hydrologic cycle with emphasis on forests and introduce the main impacts of Fennoscandic forestry on water availability and water quality. Then, we consider whether the WF correctly represents the principles of the hydrologic cycle and appropriately accounts for forest sector water use and water impacts. Finally, we consider water footprinting in general and propose necessary improvements so that it could become a robust tool for addressing water use and water impacts of forestry and forest-based products.

## Background

### Hydrologic Cycle and Forests

**Fig. 1:**
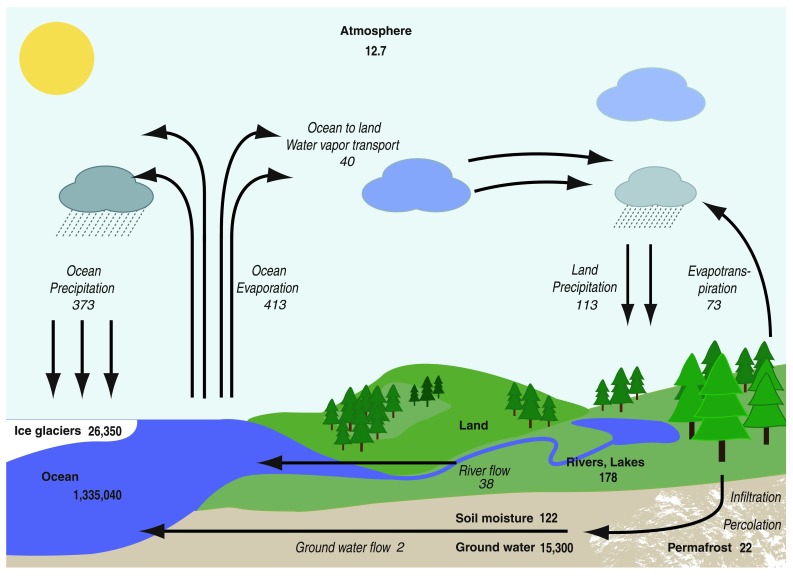
Schematic of the global hydrologic cycle. The reservoirs (in 10^3^ km^3^) are shown in *bold* and flows (10^3^ km^3^ a^−1^) in *italic*. The numbers are based on Trenberth et al. (2007) while slightly different values can be found elsewhere. Partitioning between river and groundwater flows is based on Zektser and Loaiciga (1993)

The global hydrologic cycle is a closed system, which consists of water stores and flows between them (Fig. 1). More than 97 % of global water resources are saline waters in the oceans. The great majority fresh water is stored in glaciers (77 %) and as blue ground water (22 %). Blue surface water and green water stocks in the root zone comprise less than 1 % of global fresh waters (Hornberger et al. 1998; Trenberth et al. 2007). These stores of water are not static as water is continuously circulating from one to another. Compared to other stores, the atmospheric storage is small and constrained by the global mean temperature. On annual scale, the global water flows through the atmosphere (ET and precipitation, *P*) exceed the size of atmospheric water storage by a factor of ~38. This means that an evapotranspired water molecule returns to Earth’s surface as *P*, either locally or to more distant areas, on average within 9–10 days (Hornberger et al. 1998; Trenberth et al. 2007). Globally, ~65 % of *P* falling on continents originates from terrestrial ET and the remaining 35 % comes from ocean evaporation (Oki and Kanae 2006). The partitioning of *P* into terrestrial and oceanic sources is spatially and temporally variable with *P* in some central or eastern parts of continents relying almost completely on terrestrial sources while in coastal areas oceanic sources typically dominate (see e.g., van der Ent et al. 2010). On an annual scale global *P* and ET balance with great accuracy.

**Fig. 2:**
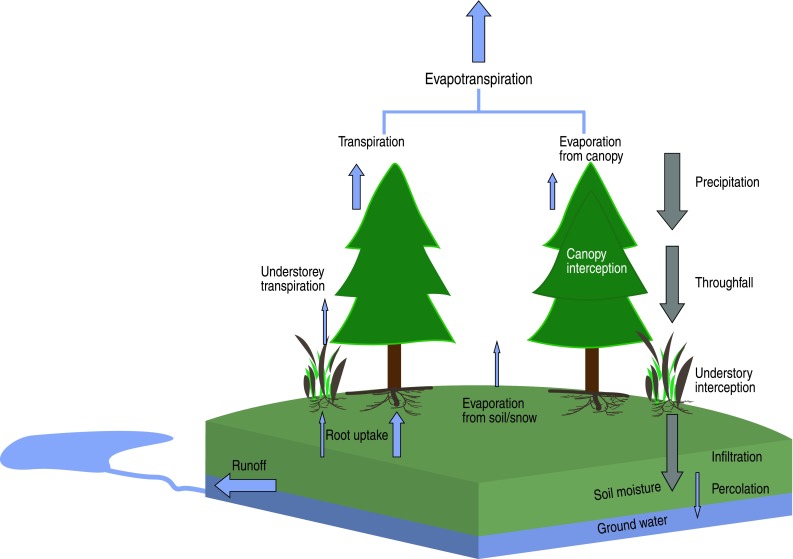
Conceptual water balance of a forest stand. The precipitation provides water input to the system which is then partitioned into different components as in Eq. 1

In a smaller scale, such as a forest catchment (Fig. 2), the water balance can be described as

1 $$ {\text{Change}}\;{\text{in}}\;{\text{water}}\;{\text{storage}} = P-{\text{ET}}-{\text{runoff}} . $$

The *P* provides green water inputs to the catchment, change in water storage refers to changes in green water stores, snow storage, blue groundwater funds/stocks, and lake volumes. Blue water flows, or runoff, are the sum of stream flow and groundwater discharges from the catchment. ET, or green water flow, is the sum of plant transpiration and evaporation from wet surfaces such as intercepted water on leaves (Fig. 2). In a forest stand, a significant fraction of *P* is intercepted by trees and understory and evaporated back to the atmosphere as non-productive green water flow. The rest infiltrates into soils increasing green water supply in the root zone. Tree roots take up green water which is transported to leaves where it is transpired to the atmosphere. Some water percolates downwards from the root zone and replenishes blue groundwater funds. Groundwater eventually discharges into surface waters or the sea, or may percolate further down to replenish fossil groundwater. Blue water runoff at the soil surface can contribute water to lakes and streams, especially on deep slopes after heavy rainfall or during snowmelt. Changes in the amount of green and blue water follow seasonal weather patterns and long-term changes in catchment water storage can occur through “mining” of fossil blue water aquifers.

Rain-fed forests only use renewable green water stores in the root zone (Fig. 2). While ET can be seen as consumption of green water from a local perspective, in a larger spatial (or longer temporal) scale it is an important part of the natural hydrologic cycle, a “service” that transports water vapor to the atmosphere and contributes to precipitation in other locations (Fig. 1; Ellison et al. 2012). Assessing human appropriation of fresh water is thus complicated by difficulties in distinguishing between water *utilization* and *consumption*. Water utilization represents flows through a system or process which are available for reuse. Consumption refers to water that is made unavailable for further use, for instance by incorporating it into a product (Koehler 2008). Because the global hydrologic cycle is a closed system these definitions are strongly dependent on the spatial and temporal scale considered.

### Impacts of Forestry on Water Cycle and Quality in Fennoscandia

The Fennoscandic landscape is dominated by forests. In Norway, Sweden and Finland forests cover between 34 and 73 % of the land area and have high importance for national and regional economies (FAO 2010). Fennoscandia is water abundant; Sweden and Finland, for example, annually extract only some 1.5–2.4 % of available freshwater resources (FAO 2012). In the region, forestry operates in areas naturally covered by forests and uses almost entirely native coniferous and deciduous tree species. Hereafter, these types of forests are referred to as semi-natural. Normal forest management in the region is final felling followed by site preparation. Felled areas are usually replanted 1–3 years after site preparation and non-commercial thinning (cleaning) is done once or twice at a stand height of 1–4 m to favor the most productive tree species. Commercial thinning is done 1–4 times per rotation period, which in Fennoscandic forestry varies between 60 and 120 years depending on tree species and site productivity. Harvested wood is used mainly for pulp production. Fertilization can be applied to enhance forest growth but is currently done on only a small fraction of managed forests (Jacobson and Pettersson 2010; Ylitalo 2011). Harvesting logging residues as well as stump removal for bioenergy has become increasingly common during the last decade (Röser et al. 2008).

Fennoscandic forests are rain-fed which means that all water contributing to ET originates from precipitation and forests in the region have no negative long-term impacts on ground water reservoirs (Rusanen et al. 2004). Forest management modifies stand structure and species composition which affect stand water balance (Table 1). Removal of trees reduces transpiration and canopy interception leading to increased infiltration and percolation. As a result, green water ET decreases, soil green water funds and local blue water runoff increase, and ground water levels may temporarily rise. Impacts of forestry on local blue water funds depend largely on the extent and intensity of forest management. Several studies have shown that effects of forest cutting become significant only when more than one-fifth of the catchment area is clear-cut, increasing annual blue water runoff by 5–40 % for a few years following felling (Haveraaen 1981; Stednick 1996, 2008; Robinson and Dupeyrat 2005; Koivusalo et al. 2006; Sørensen et al. 2009; Ben-Hur et al. 2011). The impacts vanish within 10–15 years after regeneration. This agrees with micrometeorological observations of ET in Southern Finland showing only ~10 % smaller summertime ET from a 5-year-old naturally regenerated clear-cut forest than from a 48- or 75-year-old Scots pine forests (Rannik et al. 2002; Pasi Kolari, unpubl.). These findings are supported by Canadian studies showing only minor ET differences between different-aged semi-natural boreal forests (Amiro et al. 2006). Effects of thinning and pre-commercial cleaning on water balance are significantly smaller and more short-lived than those of final felling because reductions in stand biomass are smaller. By making the runoff response to precipitation and snow melt more rapid compared to pristine peatlands, forest drainage has had the strongest hydrologic impact of forestry in the Fennoscandia. In Finland and Sweden, ~8 Mha of peatlands have been drained for forestry that has significantly increased tree growth (Paavilainen and Päivänen 1995). Peatland drainage has ceased in the 1980’s and ditch maintenance, carried out annually on some 60 000–80 000 ha (<0.3 % of forest area) in Finland, has only minor impacts on runoff (Koivusalo et al. 2008).

**Table 1:** The impacts of forestry operations in Fennoscandia on catchment water balance, the excess load of elements into water courses, and the quality of ground water. ET is evapotranspiration and *Q* runoff. Water quality impacts on surface waters are shown for suspended solids (erosion) and leaching of nitrogen, phosphorus, base cations (Ca, K, Mg), and mercury. Increases compared to the situation before operations are indicated by positive and decreases by negative sign. Data on impacts of commercial thinning on excess load into water courses does not exist

Forestry operation	Impact on catchment water balance		Excess load into water courses^a^					Impact on ground water^b^
	ET	*Q*	Suspended solids	Nitrogen	Phosphorus	Base cations	Mercury	Nitrate concentrations
Final felling (including site preparation)	−	+	+	+	+	+	+	+
Commercial thinning	±	±						
Ditch network maintenance on peatlands	+ (long term)	+ (short term) − (long-term)	+	±	+	+		
Nitrogen fertilization	+	–		+		+		

^a^Ahtiainen and Huttunen (1999), Finér et al. (2010), Joensuu (2002), Kenttämies and Haapanen (2006), Porvari et al. (2003), Ring et al. (2008), Rosén et al. (1996), Saura et al. (1995)

^b^Mannerkoski et al. (2005), Rusanen et al. (2004)

Although data on quantitative water balance impacts of forestry in Fennoscandic semi-natural forests is limited and generalizations are difficult to make due to large spatial variability in climatic factors, soil conditions, and topography, there is no evidence that forestry has had any impact on water availability. This is because impacts of clear-cutting on the water balance are short-lived and annually only a small fraction (~0.8 % in Sweden and Finland) of the forest area is clear-cut. The volume of standing timber has increased from the 1920’s by ~80 % in Sweden and by ~60 % in Finland (Ylitalo 2011) but this has not visibly altered blue water runoff availability. During the last century there has been a small increasing trend in river runoff in Sweden (Lindström and Bergström 2004), in line with the positive precipitation trend in Fennoscandia (IPCC 2001). In addition, Buttle and Metcalfe (2000) found no definitive impacts of forest harvesting on large river runoffs in northeastern Ontario, Canada.

In the water-abundant Fennoscandia, impacts of forest management on water availability are marginal and the main impact of forestry is instead on surface water quality. In general, forest ecosystems improve the quality of surface and ground water by filtering nutrients and toxic substances deposited from the atmosphere and reducing erosion and sediment transport (Brauman et al. 2007; Neary et al. 2009). In Fennoscandia, most blue ground water funds that provide drinking water are located on forest areas. Managing forests for wood production can deteriorate water quality by increasing nutrient leaching and erosion (Kreutzweiser et al. 2008). Forest regeneration operations, forest drainage, and forest fertilization may increase element load into water courses (Table 1). The increased export of nutrients after final felling is mainly caused by cessation of water and nutrient uptake by trees (Lauren et al. 2005; Löfgren et al. 2009) and it peaks within 3–5 years after cutting and lasts for 5–15 years (Rosén et al. 1996; Ahtiainen and Huttunen 1999; Ring et al. 2008; Futter et al. 2010). Ditch maintenance can increase sediment and element export for several years (Åström et al. 2002, 2005; Finér et al. 2010). Increased loads can lead to eutrophication, siltation, and other changes in aquatic ecosystems (Åström et al. 2001). Export loads are reduced in forestry through a variety of techniques including buffer zones, infiltration areas, and sedimentation ponds and pits (Joensuu 2002; Gundersen et al. 2010).

## The Water Footprint Network WF method

The WF of the Water Footprint Network purports to measure human appropriation of fresh water resources and is aimed toward a variety of goals including identification of business, process or product level water consumption, and promoting sustainable use of water resources (Hoekstra et al. 2011). When applied at a product level, the WF provides an inventory of water consumption throughout a product life cycle (the virtual water content). In the WF, water consumption is normally determined for a single catchment or a river basin, although Hoekstra et al. (2011) suggest that the method can be used at any scale. As an example of wood-based products, van Oel and Hoekstra (2010, 2012) suggest that the water footprint of paper should include green water consumption during raw material production (forestry) as well as green and blue water consumption in pulp mills and supply chains of non-wood products. Accordingly, the total WF for one unit of wood-based product such as paper (p) is

2 $$ {\text{WF}}({\text{p}}) = {\text{WF}}_{\text{forestry}} ({\text{p}}) + {\text{WF}}_{\text{industry}} ({\text{p}}). $$

Here, the WF_forestry_(p) [m^3^ (water) ton^−1^ (paper)] includes green water ET and water embedded in raw biomass and be calculated as follows:

3 $$ {\text{WF}}_{\text{forestry}} ({\text{p}}) = \left( {\frac{{{\text{ET}} + (Y \times f_{\text{water}} )}}{Y}} \right)f_{\text{paper}} \times f_{\text{value}} \times \left( {1 - f_{\text{recycl}} } \right). $$

Here, ET [m^3^ (water) ha^−1^ a^−1^] and *Y* [m^3^ (wood) ha^−1^ a^−1^] represent average annual forest ET and growth rate from where raw material is sourced, *f*_water_ [m^3^ (water) m^−3^ (wood)] is the relative volumetric water content of fresh wood, *f*_paper_ [m^3^(wood) ton^−1^ (paper)] is the paper-to-wood conversion efficiency, and *f*_recycl_ (–) is the fraction of raw material derived from recycled fibers. The *f*_value_ (–) term is a multiplier describing the value of the managed forest as a source for wood relative to all ecosystem services provided by the forest. The WF_industry_(p) includes green and blue water consumption and gray water footprint at pulp mills and in the supply chains of non-wood materials, energy, etc. The blue water footprint is determined as the difference between blue water extraction and return flows to the specific catchment or river basin where production takes place. The gray water footprint is estimated as the volume of fresh water needed to dilute the largest effluent concentration in waste water to a maximum acceptable level in the receiving water body, as defined by local water quality standards (for details, see Hoekstra et al. 2011; UPM 2011).

Recent case studies for paper products (van Oel and Hoekstra 2010; StoraEnso 2011; UPM 2011) indicate that the majority (60–99.9 %) of the WF for paper arises from green water ET during wood production. Studies also show that gray water footprints of pulp mills can form a significant fraction (39 %) of the WF (UPM 2011) while blue water use, representing mainly blue water evaporated during production processes, is nearly negligible (<1 %).

## Discussion

### Concerns of the WF of Forests and Forest Products

The aims of the WF are broad and ambitious. However, when applied to forests and forest products such as paper the results leave room for interpretation and tend to raise more questions than they answer. A survey of the WF case studies (van Oel and Hoekstra 2010, 2012; StoraEnso 2011; UPM 2011) gives rise to a number of questions and concerns:

*First*, forest management and wood production in many areas, including Fennoscandia, is directed to semi-natural forests that would exist and utilize green water resources (Fig. 2) regardless of human management. Therefore, the assertion that ET from managed forests is a human appropriation of water is troubling. Currently, ET from managed forests is interpreted as a green water footprint of forestry instead of seeing it as part of a natural hydrologic cycle (Fig. 1). Failing to consider ET as a central component in the hydrologic cycle may result in inappropriate or incorrect estimates of forest sector water resource impacts.

*Second*, sustainably managed forests, especially semi-natural forests, provide several valuable ecosystem services. They maintain biodiversity and provide clean drinking water, rural employment, recreation, and a source of food (Hein et al. 2006). In addition, managed forests are major carbon stores (e.g., Jackson et al. 2005). Determining the value of each of these ecosystem services is, however, difficult and depends on cultural and regional context which complicates the interpretation of WF results.

*Third*, the use of recycled paper as a source of pulp is assumed to reduce the water footprint but the import and export patterns of paper are not realistically considered. According to van Oel and Hoekstra (2010, 2012), the WF of paper produced in, e.g., central Europe is significantly reduced because large paper import and consumption relative to local production leads to high *f*_recycl_. The *f*_recycl_ is determined as the fraction of paper produced from recycled pulp in a specific country. Consequently, *f*_recycl_ is strongly impacted by import–export patterns and it cannot be attributed to national paper recycling rates. For instance, van Oel and Hoekstra (2012) report *f*_recycl_ equal to 5 % for Finland although 71 % of paper consumed in Finland is recycled (EEA 2010). Thus, the suggestion that large potential for WF reduction exists in countries having small *f*_recycl_ (van Oel and Hoekstra 2012) is not realistic. Taken literally, the current WF model leads to the perverse conclusion that companies operating in areas with abundant resources of water, such as Finland and Sweden, should import recycled paper to reduce their local water footprints.

*Fourth*, the water quality impacts of forestry and forest industry are not well considered. The WF includes a gray water component as the total water volume needed to dilute pollutant or nutrient emissions (Hoekstra et al. 2011). In its present form, the gray water footprint does not provide a credible measure of *degradation*, i.e., the amount of polluted water that is made unavailable for further human use or natural ecosystems in the catchment. Thus, comparability of gray with blue and green water footprints, which represent physical water use, is questionable. The gray water concept contains no information about effects of emissions on downstream ecosystem service delivery. Furthermore, the gray water footprint of forestry has not been satisfactorily addressed (van Oel and Hoekstra 2010; StoraEnso 2011; UPM 2011). This is a major issue especially in water-abundant regions, such as Fennoscandia, where the most important water use impacts of forestry (and the forest sector as a whole) are related to water quality rather than availability. Finally, as revealed in the UPM (2011) study, emissions to sea/brackish water (regardless of the amount of pollutants contained) are not counted as gray water which may be a significant limitation when WF results are used in environmental sustainability assessment.

*Fifth*, WF results are highly dependent on the spatial and temporal boundaries set for the water use inventory. A practical example can be found in the UPM (2011) study where a pulp mill located next to an intersection of two rivers has a large blue water footprint when it takes its process water from one and discharges it into another river basin. While this correctly reflects the situation from a local water resource management perspective, providing such scale-dependent results as part of, e.g., a product level WF is not feasible and may lead to misinterpretation.

*Sixth*, the WF does not directly consider local conditions or address impacts and sustainability of water use. In a sustainability context, it is widely recognized that volumetric measures mean very little if they are not referenced to local water availability or environmental vulnerability (Watson 2008; Milá i Canals et al. 2009; Riddout et al. 2009; Jeswani and Azapagic 2011; Riddout and Pfister 2010). For contextualization of water use, the WF method includes a separate *water use sustainability assessment* phase. Hoekstra et al. (2011) suggest that to be sustainable, the green water footprint should not exceed available green water resources but do not provide a robust method to estimate green water availability. However, rain-fed forests can never use more green water than is available for their root uptake. Some subtropical monoculture plantations may access blue ground water funds through their deep root systems (or may be irrigated) but according to the WF methodology this should be accounted as blue water use. To be sustainable, the blue water footprint should remain below local blue water availability, defined as the difference between stream runoff and the minimum flow required by the natural environment (Hoekstra et al. 2011). While this is logical when total blue water use within a catchment or a river basin is considered, it does not allow for addressing sustainability of water use of a single process, a product or “fair share” of water resources of a single company. Gray water sustainability assessments suffer from the same problem as they are deemed sustainable whenever gray water flows are below actual blue water runoff in receiving waters. The above-mentioned methods do not provide a robust tool for assessing sustainability of water use in paper production (UPM 2011).

### The WF and the Hydrologic Cycle

We believe a major reason why WF results for forest-based products are opaque is related to the fact that spatial and temporal scales influence whether water use is understood as consumption or utilization. In this respect, a comparison between carbon and water footprints is illuminating. The carbon footprint measures the climate warming potential of (non-water) greenhouse gas emissions throughout the life cycle, in units of CO_2_ equivalents. Because greenhouse gases have long atmospheric life-times (typically from tens to hundreds of years) their emissions accumulate in the atmosphere. Emissions from different parts of the globe have nearly equal impact on climate warming thus it is possible to calculate carbon footprints by simply accumulating emissions over product life cycles. The same simplifications do not, however, hold for fresh water and it is therefore more difficult to accommodate water use and its impacts into a single indicator (e.g., Riddout et al. 2009; Wichelns 2011). Because fresh water is a circulating resource (“Hydrologic cycle and forests” section, Fig. 1), water use that appears to be water consumption from the perspective of one river basin (or in a short time scale) is water utilization if fresh water is considered as a global resource (or over long time periods). The water consumed locally during a production step does not leave the natural hydrologic cycle but rapidly returns as *P* to land areas or oceans and is available for reuse. This means that water consumption in different stages of a product life cycle is not additive and it is physically incorrect to calculate water footprints of products with complex, spatially disconnected production chains by simply aggregating local water consumption determined at catchment or river basin level. This is, however, the way in which the WF’s of products are currently calculated. This problem becomes especially clear when green water ET of long-rotation semi-natural forests are considered.

### How the Water Footprints Could be Improved for Forestry and Forest-Based Products?

A major problem in the WF and other water footprinting methods is the lack of clarity of the question addressed. It is uncertain whether water footprints should be measures of human appropriation of water resources, water resource management tools, water use efficiency measures, or water use sustainability indicators. This is a major concern as it is unlikely that a single method could reliably address all these issues. In its present format, the WF of the Water Footprint Network does not provide a method for assessing human appropriation of fresh water in forestry or to address sustainability of forest sector water use. To demonstrate this and to suggest improvements for water footprint methodologies, we use water use and water use impacts of the Fennoscandic forestry (“Impacts of forestry on water cycle and quality in Nordic/Baltic region” section) as a practical case example. Most of the suggestions can, however, be generalized beyond this specific case and geographical location.

First, if the aim of water footprinting is to measure human appropriation of water resources, all ET from managed semi-natural forests should not be attributed as a green water footprint (“Hydrologic cycle and forests” section, Fig. 2). It would be more logical to consider potential differences in water use between managed and unmanaged forests. When considering this “net green water use”, it is, however, disputable whether one should account for short-term or small-scale effects of forestry operations or changes in the forest water balance over a whole rotation cycle or over larger areas. Considering human appropriation, Riddout and Pfister (2010) suggest that green water use should be included in water footprints of agricultural products (in their case they weighted the water consumption by a local water consumption-to-renewal ratio) only if green water use creates changes in local blue water flows or funds. However, they did not provide any baseline land use to which ET changes should be compared and finally neglect it. The reasoning was that most agricultural systems are rain-fed and have no negative impacts on blue water resources. The same is valid for forests in the Fennoscandia as there is no evidence that forestry has a significant impact on blue water availability (“Impacts of forestry on water cycle and quality in Nordic/Baltic region” section). Therefore, we suggest ET of semi-natural forests be excluded from the water footprint as a naturally occurring phenomenon.

Second, the goal of water footprinting could be to address and compare water use efficiency. This is one of the main aims of the WF and its results are represented in units that can be interpreted as water use efficiency, the amount of water consumed per unit of product (Hoekstra et al. 2011). As discussed in “The WF and the hydrologic cycle” section, water consumption volumes should not be aggregated over the production chain. Therefore, the water use efficiency of each production step should be considered and communicated separately. We recognize that providing a single aggregate number may be preferred for simplicity and the sake of awareness raising but emphasize that it tends to create interpretation problems. It has not, for instance, been possible to trace and compare water use efficiency in different steps of paper production or between different products or companies (e.g., case studies of StoraEnso 2011; UPM 2011). In the case of water use efficiency of forest-based products it would be essential to consider and report blue water use in different production steps separately. The process blue water efficiency is a clearly defined and routinely measured quantity (e.g., Wiegand et al. 2011) that also has the potential to form an important competitive asset among companies and thus promote water efficient processes. Also, pursuing greater blue water use efficiency can in general be considered positive due to linkages among water use efficiency, energy efficiency, and greenhouse gas emissions.

Promoting greater water use efficiency in the forestry part of the production chain is, however, a much more complex issue and it is not necessarily beneficial to aim for greater green water use efficiency in forestry. The green water footprints of fresh wood (essentially equal to annual ET/annual yield) provided by van Oel and Hoekstra (2012) indicate that for example the green water footprint of Scots pine wood grown in Finnish boreal forests would be 592 m^3^ ton^−1^, 1.5–3 times that of Eucalypt wood produced in Australia (415–438 m^3^ ton^−1^), Portugal (314 m^3^ ton^−1^), or Brazil (214–233 m^3^ ton^−1^). Furthermore, van Oel and Hoekstra (2012) suggest that the global water footprint of paper can be reduced by sourcing wood production to areas and species that are water efficient, i.e., have lowest green water footprints. This would suggest that, for instance, Mediterranean and sub-tropical regions, where water scarcity problems are not unusual, would be more appropriate locations for producing wood than semi-natural forests in the water-abundant Fennoscandia. However, if the aim is to sustainably manage local and global water resources, it is crucial that water use efficiency in forestry is considered with reference to alternative productive land uses. In this sense, wood production is water efficient in water-abundant regions, even if forest growth is slower due to a harsher climate, less fertile soils, and less productive (native) species adapted to these conditions.

Currently, the WF and especially the way its results are reported incorrectly penalizes Fennoscandic semi-natural forests for their low growth-to-ET-ratio although the water use from managed forests in the region does not significantly differ from that of unmanaged forests and does not threaten water resources. Similar risks could easily emerge if forest-based bioenergy, often a side or a supplementary product in semi-natural forestry, were to be contrasted to agricultural bioenergy produced in semi-arid regions. Providing and communicating green water footprints in their current format creates risks for misunderstandings that may have harmful effects both on where and how forests are managed and also on the sustainable use of water resources.

Third, if the water footprints are interpreted as measures of sustainability, it is essential that water use is considered in the context of local conditions. As impacts of water use on water quality and availability are typically local or regional, defining a water footprint as a spatially explicit impact potential could provide a more robust way to address responsible water use and management. As there is hardly any relationship between water scarcity in one region and water consumption in another (e.g., Riddout et al. 2009; Wichelns 2011), the sustainable use of global water resources to a large extent equals minimizing negative impacts of water use. In water-abundant areas, such as Fennoscandia, the large amounts of water used by forests and forest industry pale in comparison to the total amount of water available as precipitation. Water use impacts will be very different for plantation forestry in semi-arid environments, where concerns about water availability and risks of negative impacts on water availability and overlapping land use impacts are more likely. Similarly to the cases of agricultural products (Riddout and Pfister 2010) and biofuels (Gerbens-Leenes et al. 2009; Hoekstra et al. 2009; Pfister and Hellweg 2009; Gheewala et al. 2011), placing the water use of forestry and forest products in the context of local water availability would provide a more realistic view of water use sustainability than reporting water footprints as they currently are in the WF.

There are a number of important developments of the water footprint toward a measure of impact potential. These weighed water use indicators address sustainability either through measures of water availability such as water stress or scarcity (e.g., Pfister and Hellweg 2009; Riddout and Pfister 2010) or through both availability and potential environmental impact (Milá i Canals et al. 2009; Pfister et al. 2009). Milá i Canals et al. (2009) considered water use impacts at a river basin level and identified two primary impact pathways: freshwater ecosystem impact and slowly recoverable or permanent depletion of freshwater resources. Changes in water availability caused by blue surface water use as well as land use changes that alter the (green) water cycle are considered to have freshwater ecosystem impacts. Freshwater depletion refers to over-exploitation of blue water funds and deposits (such as fossil ground water) that creates slowly recovering depletion of these water stores. In the “Impacts of forestry on water cycle and quality in Nordic/Baltic region” section, it is shown that green water use in Fennoscandic forestry does not have a notable effect on blue water resources, either on groundwater funds or surface water flows. Consequently, it is fair to suggest that green water use of these forests can be removed from water footprinting if the aim is to address sustainability in terms of water availability.

Currently, weighted water use indicators mainly use only water availability measures, such as water use to replenishment ratios, as their characterization factors. Because forest sector water-related impacts, at least in the boreal region, often occur through mechanisms that are unrelated to water availability measures, additional characterization factors would be needed to describe water quality impacts. Recent development of life-cycle impact assessment (LCA) methods to better incorporate land occupation and land use change impacts could provide a way to address both positive impacts of forests as a land use and potential negative impacts of forestry on fresh water resources (Milá i Canals et al. 2012). Saad et al. (2011) propose LCA characterization factors for land occupation impacts on freshwater regulation potential (i.e., impact on blue water availability), erosion regulation potential, and water purification potential (i.e., impact of land use on blue water quality). Another possibility could be to include surface water quality impacts of forestry and forest industry through eutrophication potential (e.g., Seppälä et al. 2004). Long-term monitoring of water quality impacts of forests and forest management in Fennoscandia can provide basic data for testing and developing these methods in the forest sector.

There are, however, several concerns about the use of weighted water use indicators at a product or company level. One is the difficulty of relating water use to environmental or social harm as there is a lack of reliable and quantitative indicators (characterization factors) to assess water use impacts (Riddout et al. 2009; Jeswani and Azapagic 2011). It is also difficult to exactly quantify impacts of a single company or a product on the environment or water resources whenever multiple users operate in the same area (Lambooy 2011). The second is the question of appropriate level of aggregation and considers geographically and temporally highly variable water availability and environmental vulnerability, which creates strong demands on both spatial and temporal resolution and accuracy of input data. The locality of water use impacts is a challenge for forest products, as raw material production is often spatially disconnected from the final production sites (e.g., pulp mills). The third problem is that, as in the case of aggregated water use efficiency metrics, interpretation of weighted water use indicators is not straightforward and without supplementary information it is impossible to distinguish whether the weighted result is primarily determined by the amount of water used or by local water availability.

### Communication About Water

The critique presented against the WF is not to diminish the importance of considering water use and making transparent the water-related impacts of forestry and the forest sector. The complexity of ecosystem–water–human interactions and the extreme variability of water use impacts make it unlikely that water use and water use impact measures could in the near future be used for quantitative comparisons of forest sector products. The international marketplace is not well-situated to fully understand and appreciate local circumstances and the importance of tradeoffs between different kinds of water use allocation (Wichelns 2010). Therefore, using highly simplified quantitative water footprints—compelling at first sight but staggering in their information content—in external communication may create a risk for misinterpretation. It may thus be advisable to provide more complete “water profiles” as proposed by Wiegand et al. (2011) and NCASI (2009, 2010). In comparison to the WF that tries to aggregate complex processes and a large amount of information into a single number, such broader water profiling should allow direct consideration of blue water use efficiency of different processes and water impacts of forestry, as well as providing measures of improvements or changes in water management practices. If the WF methodology was to be revised and its results presented differently, as previously discussed, it could form a part of such water profiling. From a communication point of view it is essential to provide guidelines and harmonize ways in which water-related issues are communicated to stakeholders, customers, and the public.

If water footprinting results are to be taken to a product level it seems that a qualitative water labeling, instead of quantitative footprint, would be a better and less error-prone option. Such a general product labeling could be based on, e.g., best available techniques and practices in water use and water protection (Hoekstra 2011), with reference to local conditions and accounting for the special characteristics of each industry. Current development of the International Water Stewardship Standard (AWS 2012) is an example of such a move toward a general producer-oriented water certificate. This type of certification resembles forest certification schemes such as FSC and PEFC that are widely accepted tools for supporting sustainable environmental management in forestry. In fact, these schemes to some extent already include good water protection practices as one part of their sustainability criteria. It can be argued that such a general water labeling does not allow direct comparison between products or companies. When forestry and forest-based products are considered, we, however, claim that such a direct comparison may not be necessary or even desirable from a sustainability perspective. As the industry to the largest extent relies on natural resources in the form of biomass, wood, fiber, and water; sustainability of water use and water resource management should be considered in a broader context accounting for linkages among water use, energy use, multi-functionality of forest ecosystems, values and socioeconomic demands. Considering forestry and the existence of forests, these include trade-offs between carbon assimilation and local stream flow (e.g., Jackson et al. 2005), conserving biodiversity, maintaining, and providing high quality drinking water and other ecosystem services (Brauman et al. 2007; Neary et al. 2009). In this regard, the LCA analyses that address sustainability in a broader sense than water footprints could be more appropriate.

## Conclusions

The water footprint (WF) developed by the Water Footprint Network is an ambitious tool aimed at measuring human appropriation of freshwater and promoting sustainable use of fresh water resources. This article has provided a thorough consideration of its applicability for forestry and forest-based products deriving examples from published case studies and known water impacts of forestry in water-abundant Fennoscandia. Most of the arguments and suggestions can, however, be generalized beyond this specific case and geographical location. We noted that aggregating local (a catchment or a river basin level) water consumption over a product life cycle is inconsistent with the principles of the hydrologic cycle and does not correctly treat water as a circulating resource. We showed that there is no evidence that ET from managed semi-natural forests in Fennoscandia would notably differ from that of unmanaged forests, and that forest management has not had a significant impact on blue water availability. Instead of considering forest ET as part of natural water cycle, the WF at present accounts all ET from managed forests as a human appropriation of green water. This may lead to serious misinterpretations of the water use and water resource impacts of forestry and forest-based products. We therefore recommend that ET of rain-fed semi-natural forests should not be part of the water footprint and that special care should be taken when evaluating and communicating water resource impacts of forest-based products and services.

We emphasize that the general goal of water footprinting and the way its results are presented needs to be clarified and methods developed accordingly to make water footprinting a tool that can also be used in environmental communication of forestry and forest-based products. In particular, if the aim of the water footprint is to provide a tool for sustainable water resource management, water use and water-related impacts should always be contextualized with local water availability and environmental sensitivity.
